# Barriers to Same-Day Pre-exposure Prophylaxis (PrEP) Implementation in Federally Funded HIV Clinics Within High-Burden Areas of the U.S.: A Coincidence Analysis

**DOI:** 10.1007/s10461-025-04898-2

**Published:** 2025-10-10

**Authors:** Alithia Zamantakis, Wilson Gomez, Lipin Lukose, Ana Michaela Pachicano, Jessica Kassanits, Anna-Sophia Katomski, Elena P. Rosenberg-Carlson, Cathleen E. Willging, LaRon E. Nelson, Tequetta Valeriano, Maile Karris, Jill S. Blumenthal, Aadia I. Rana, Russell A. Brewer, Alison Hamilton, Christopher Kemp, Debbie Humphries, Nanette D. Benbow, Uyen Kao, Sung-Jae Lee, Joyce L. Jones, Sheree R. Schwartz, Laura K. Beres, Joseph G. Rosen

**Affiliations:** 1 Institute for Sexual and Gender Minority Health and Wellbeing, Northwestern University, Chicago, IL, USA; 2 Department of Medical Social Sciences, Feinberg School of Medicine, Northwestern University, Chicago, IL, USA; 3 Department of Epidemiology, Bloomberg School of Public Health, Johns Hopkins University, Baltimore, MD, USA; 4 Center for HIV Identification, Prevention, and Treatment Services, David Geffen School of Medicine, University of California Los Angeles, Los Angeles, CA, USA; 5 Pacific Institute for Research and Evaluation, Albuquerque, NM, USA; 6 Center for Methods in Implementation and Prevention Science, School of Nursing, Yale University, New Haven, CT, USA; 7 Division of Infectious Diseases, School of Medicine, University of California San Diego, San Diego, CA, USA; 8 Division of Infectious Diseases, Heersink School of Medicine, University of Alabama at Birmingham, Birmingham, AL, USA; 9 Chicago Center for HIV Elimination, University of Chicago, Chicago, IL, USA; 10 Department of Psychiatry and Biobehavioral Sciences, UCLA David Geffen School of Medicine, Los Angeles, CA, USA; 11 School of Public Health, Yale University, New Haven, CT, USA; 12 Department of Psychiatry and Behavioral Sciences, Feinberg School of Medicine, Northwestern University, Chicago, IL, USA; 13 Department of Psychiatry and Biobehavioral Sciences, David Geffen School of Medicine, University of California Los Angeles, Los Angeles, CA, USA; 14 Department of Epidemiology, Fielding School of Public Health, University of California Los Angeles, Los Angeles, CA, USA; 15 Division of Infectious Diseases, School of Medicine, Johns Hopkins University, Baltimore, MD, USA; 16 Department of International Health, Bloomberg School of Public Health, Johns Hopkins University, Baltimore, MD, USA; 17 Division of General Internal Medicine, Rhode Island Hospital, Providence, RI, USA; 18 Department of Medicine, The Warren Alpert Medical School, Brown University, Providence, RI, USA

**Keywords:** Same-day PrEP, Biomedical HIV prevention, Configurational analysis, Ending the HIV epidemic, United states, Implementation science

## Abstract

**Background:**

Same-day pre-exposure prophylaxis (PrEP) for HIV prevention is a novel tool to increase biomedical prevention access and uptake. Although prior research has identified same-day PrEP as feasible and acceptable, improved understanding of key barriers and facilitators to implementation is needed to optimize impact.

**Methods:**

The Network for Implementation Science in HIV (NISH) examined the implementation of same-day PrEP across 42 Ryan White Part A-D-funded organizations in seven Ending the U.S. HIV Epidemic Initiative priority jurisdictions. We used crisp-set coincidence analysis with six conditions to identify key barriers explaining delayed implementation of same-day PrEP among 38 clinical organizations representing 137 individual clinics where same-day PrEP services were available currently or in the future.

**Results:**

Our final model explained ~ 67% of clinical organizations not currently implementing same-day PrEP (*n* = 8), with a consistency of 80%. This model identified three pathways to non-implementation of same-day PrEP: (1) insurance coverage as a top barrier *and* insufficient staffing resources as a top barrier; (2) no onsite pharmacy as a top barrier; *or* (3) provider reticence with same-day PrEP prescribing and insurance coverage as top barriers, *and* patient demand/(dis)interest in same-day PrEP not being ranked as a top barrier.

**Conclusion:**

The three identified pathways to delayed implementation of same-day PrEP aid researchers and providers in reverse mapping strategies to enhance and scale up the implementation of same-day PrEP in federally funded clinics in the U.S. Such strategies may include federal and state-level policy changes to expand funding for PrEP coverage, pharmacist-led same-day PrEP implementation, or mail delivery of PrEP prescriptions.

## Introduction

Approximately 1.2 million people in the United States are living with HIV, 13% of whom are unaware of their HIV status [1]. According to the U.S. Centers for Disease Control and Prevention (CDC), new HIV diagnoses in the U.S. decreased by 12% from 2018 (36,200 cases) to 2022 (31,800 cases), attributed in part to the scale-up of combination HIV prevention interventions including pre-exposure prophylaxis (PrEP)—an effective medication that reduces the risk of sexually mediated HIV acquisition [1]. However, research indicates that populations who shoulder disproportionate HIV burdens—particularly across racial/ethnic, age, and geographic strata—remain underrepresented among PrEP users [2–5]. For instance, HIV incidence rates among Black cisgender men who have sex with men (MSM) are 10 times greater than among non-Latine white MSM; however, Black MSM are half as likely as non-Latine white MSM to use PrEP [6, 7]. Enhancing PrEP implementation in priority populations and geographies is essential for achieving the Ending the HIV Epidemic (EHE) Initiative’s goal of a 90% reduction in new HIV infections by 2030 [8].

Existing literature indicates numerous barriers to uptake and adherence along the PrEP care continuum [9]. Barriers include difficulties accessing a provider who prescribes PrEP [10–12], challenges of attending multiple appointments [13, 14], and unmet health-related social needs (e.g., suboptimal transportation access) [14, 15]. Accordingly, an important tool to increase PrEP uptake includes same-day PrEP initiation [16, 17]. Listed as a recommended approach in the recently updated CDC guidance for HIV prevention, same-day PrEP initiation is an evidence-based practice. Initiation of same-day PrEP immediately follows an HIV-negative preliminary diagnosis with a same-day prescription for four weeks of oral PrEP or same-day initiation of long-acting injectable (LAI) PrEP [18]. The CDC guidelines highlight that same-day PrEP may be particularly appropriate for individuals with work constraints or factors rendering them more vulnerable to HIV acquisition (e.g., substance use), enabling providers to reach individuals at the time of rapid HIV testing, rather than retroactively linking them to PrEP care following confirmatory testing [9].

Prior research on same-day PrEP has pointed to some barriers and facilitators of implementation, including the potential importance of patient navigation for same-day PrEP initiation [19–22]. This approach educates individuals about PrEP, provides them with financial navigation assistance, and links patients to other services to address health-related social needs [19–22]. Investigators have also identified patient-provider trust as an important facilitator of same-day PrEP implementation [20, 22]. Building a trusting relationship through open communication, addressing patient concerns, and counseling patients on the benefits and potential side-effects of PrEP have been identified by individuals who use PrEP as “core components” of a same-day PrEP process [20]. Implementing same-day PrEP outside a clinic (e.g., in a community pharmacy), reducing required paperwork, decreasing visit time, and/or making the workflow more efficient also facilitates same-day PrEP implementation [17, 23]. This growing body of research has identified patient and provider-level barriers through qualitative analyses, but, to our knowledge, no study has attempted to discern the configurations of barriers that hinder systems-level implementation of same-day PrEP. Moreover, a burgeoning body of evidence indicates that the “success” of PrEP implementation hinges upon the presence (or conversely absence) of myriad forces (e.g., provider competencies, specialized insurance schemes, supplemental funding and human resources [24–26]); the imperative to identify synergistic effects of these determinants on same-day PrEP implementation has been widely discussed but insufficiently executed in the extant scholarship.

As same-day PrEP prescription is a relatively nascent approach in U.S. clinical settings, additional research is needed to identify which factors operate synergistically to facilitate or, conversely, delay implementation of same-day PrEP. Identifying these barriers to implementation will aid researchers and implementers in identifying and developing strategies to accelerate same-day PrEP adoption and equitable implementation. Accordingly, we used coincidence analysis (CNA) to identify the key barriers to same-day PrEP implementation in a multi-jurisdictional sample of clinics delivering HIV care and treatment services funded by the Ryan White HIV/AIDS Program. CNA is a novel analytic approach that uses Boolean algebra to identify the combination(s) of explanatory variables (referred to as factors) that necessarily and sufficiently explain an outcome [27, 28]. CNA identifies factors (or variables) that are difference-makers; that is, their presence or absence has a measurable impact on the presence or absence of the outcome [28]. CNA is comparatively robust in small samples and in cases of equifinality (i.e., when multiple combinations of factors, or “pathways,” may be associated with or explain an outcome) [29]. When CNA explores the necessary and sufficient factors to produce an outcome, CNA identifies “pathways” to an outcome may differ across cases (e.g., units of analysis). As such, CNA helps to identify whether, while one set of factors may lead to an observed outcome for some clinics, there may be a different set of factors that explain the presence or absence of this outcome for other clinics. CNA’s ability to examine context-specific, discrete pathways to an outcome is informative for implementation researchers aiming to identify implementation strategies, or methods, processes, and practices to enhance the effectiveness and increase adoption of same-day PrEP implementation in additional clinics and jurisdictions [30].

## Methods

### Study Design and Procedures

Data were derived from a multi-site study led by the Network for Implementation Science in HIV (NISH), comprising a collaboration led by the HIV Implementation Science Coordination Initiative (ISCI) at Northwestern University, the University of Chicago, and five university-based Regional Consultation Hubs (Johns Hopkins University, University of Alabama at Birmingham, University of California—Los Angeles, University of California—San Diego, Yale University) across the U.S [24, 31, 32]. The objective of the parent implementation planning study was to characterize the adoption and determinants of rapid antiretroviral therapy (ART) and same-day PrEP at clinics funded by the Ryan White HIV/AIDS Program (RWHAP) for outpatient/ambulatory care services in seven of the fifty-seven EHE jurisdictions: Alabama (all 67 counties); Baltimore (County of Baltimore City), Maryland; Chicago (Cook County), Illinois; Dallas (Dallas County), Texas; Fort Worth (Tarrant County), Texas; Los Angeles (Los Angeles County), California; and San Diego (San Diego County), California [8]. The seven EHE jurisdictions were selected due to their alignment with ISCI and the Regional Consultation Hubs. Yale University is not within an EHE jurisdiction, but their Regional Consultation Hub includes faculty members located in Dallas/Fort Worth, Texas.

Between February 2023 and April 2024, an online survey was circulated with administrators or leadership of eligible RWHAP-funded clinical organizations across the seven EHE jurisdictions to characterize the magnitude and determinants of same-day PrEP implementation at the clinic level. The Health Resources and Services Administration (HRSA) clinic locator was used to construct a sampling frame of clinical organizations meeting the following inclusion criteria: (1) providing HIV care/treatment services in one of seven EHE jurisdictions as of January 2023 and (2) receiving funding from RWHAP Parts A, B, C, or D [33].

After determining a list of eligible clinical organizations, study personnel identified senior clinicians and administrators to introduce the study objectives and circulate a weblink to a 34-question survey eliciting experiences with rapid ART and same-day PrEP implementation. Administrators or leadership were prompted to complete the survey, hosted on Northwestern University’s REDCap^®^ electronic data capture platform, for their clinical organization within one month from the date of the initial invitation to participate [34, 35]. Recruitment of clinical organizations was rolling to accommodate staggered review timelines and approvals from locally governing ethics review boards within each EHE jurisdiction. Clinical organizations completing the survey received a locally appropriate food basket/item intended for consumption by the organization’s staff, limited to $100–200 per clinical organization.

### Measures

As noted, we sought to identify the configurations of barriers that explain delayed implementation of same-day PrEP (measured dichotomously as (1) current availability or (2) intent to offer same-day PrEP within the next 12 months [i.e., delayed implementation]). Respondents were prompted to rank three of the following 13 items that presented the most substantial challenges to same-day PrEP implementation within their respective practice contexts: (1) leadership buy-in and support barriers; (2) provider reticence to prescribe without full complement of laboratory results; (3) provider disinterest; (4) insufficient staff awareness, knowledge, and training; (5) insurance coverage barriers; (6) insufficient staffing resources; (7) existing clinic workflows barriers; (8) competing agency priorities; (9) perceived (dis)interest from clients; (10) insufficient support for client psychosocial needs; (11) lack of onsite medications and/or collaborating pharmacy; (12) lack of onsite phlebotomy and/or laboratory services; and 13) lack of implementation protocols or guidelines. Barriers were deemed present for each organization when specific items were ranked as a top three barrier or absent as a leading barrier if unselected as one of the top three barriers. A full list of items can be found in Table 1.

### Analysis

#### Calibration

We used crisp-set CNA (i.e., factors, [or variables], and the outcome were dichotomized; 0 = *absent*, 1 = *present*), using the CNA package 3.5.3.4 in R software to identify which implementation barriers (together with or separate from the presence or absence of other barriers) explained the *delayed implementation* of same-day PrEP among RWHAP-funded clinics (i.e., those not currently implementing but intending to do so within the next 12 months) [36, 37]. We began by calculating descriptive statistics for all survey items, ascertaining whether itemized implementation barriers exhibited sufficient variability for inclusion in CNA [38].

#### Factor Selection

We used the minimally sufficient condition (“msc”) approach to determine which factors to include in the analysis based on coverage and consistency [36, 39–41]. Coverage, ranging from 0 to 1, refers to the proportion of cases with the outcome explained by a CNA model (i.e., a set of factors) [29]. Consistency, also ranging from 0 to 1, refers to how reliably a CNA model explains the outcome [42]. Using the “msc” approach, three study team members (az, WG, JGR) independently assessed which factors were most commonly present in factor configurations.

We used both content and case knowledge, as well as the means and medians of the frequency of factor’s presence in configurations as thresholds to determine the inclusion of each factor. The use of means and medians of frequency is a more novel approach we used to balance our own subjective knowledge with a more concrete criterion. We also prioritized factors in configurations with lower complexity, both to reduce the potential for redundancy and as, in implementation science, the less complex a configuration as, the more feasible it will be to implement [39]. The following six factors were retained, based on the frequency of presence above the mean (4.46) and the median (4): (1) provider reticence to prescribe without full complement of laboratory results as a top barrier, (2) insurance coverage as a top barrier, (3) insufficient availability of staffing resources as a top barrier, (4) perceived demand or (dis)interest from clients as a top barrier, (5) lack of onsite phlebotomy and/or laboratory services as a top barrier, and (6) lack of implementation protocols or guidelines as a top barrier.

#### CNA

We conducted a CNA with consistency and coverage thresholds of 95%, then progressively reducing coverage and consistency by 5% until an adequate model was identified. Since non-implementation of same-day PrEP was present in only 32% of the cases, we could not identify models above a threshold of 75% (a common CNA threshold in the extant literature) [39, 42–44]. We used prevalence-adjusted consistency and prevalence-adjusted contrapositive coverage thresholds to account for an imbalance of the presence and absence of the outcome, using the R package CNA 3.5.3.4.tar.gz [38]. We began with prevalence-adjusted consistency and prevalence-adjusted contrapositive coverage at lowered thresholds of 80%, then decreased each by 5% until adequate models were identified [38]. Overall, 33 candidate models were identified and independently assessed by three study team members (az, WG, JGR) based on consistency, coverage, and complexity. Five candidate models were selected and presented to study investigators during a 3-day hybrid meeting that accommodated face-to-face and virtual participation among the investigative team. Three final models were then selected. Next, we presented the final three candidate models to study principal investigators (PI), members of the study leadership with intimate knowledge of clinical implementation of same-day PrEP, to further guide model selection.

Data were managed and descriptively analyzed in Stata/MP 18.0, and coincidence analysis was implemented in RStudio^®^ version 2024.04.2 + 764 (see Online Resource 2 for our complete R script) [37, 45].

### Ethics

Northwestern University submitted a single institutional review board (IRB) protocol through Advarra (Pro00066355), which deemed the protocol exempt from further human subjects research oversight. Each site also submitted a local IRB application through their respective institutions; local IRBs either determined the protocol to be exempt, reviewed and approved it, or entered into a reliance agreement with Northwestern University. A data use agreement (DUA) was also executed between Northwestern University and the University of Chicago.

## Results

### Clinic Characteristics

Of the 83 clinical organizations invited to participate, a total of 42 entities completed the survey (response rate: 50.6%), 38 of which reported current (*N* = 26) or prospective (*N* = 12) same-day PrEP implementation (see Table 2). The 38 clinical organizations reported current or prospective implementation represented a total of 137 clinics. Most of the 137 clinics included federally qualified health centers [FQHCs; 37% (*n* = 14)], followed by academic-affiliated health centers [26.0% (*n* = 10)]. The 137 clinics covered primarily urban geographies [66.0% (*n* = 25)] in Los Angeles (21.0%), Chicago (21.0%), San Diego (15.8%), Alabama (15.8%), Baltimore (15.8%), and Dallas Forth Worth/Tarrant County (10.5%). A median of 23 (*interquartile range*: 17, 50) employees per clinical organization reported providing HIV-related services, 7 (*interquartile range*: 5, 14) of whom were licensed to prescribe ART or PrEP. The clinical organizations reported providing PrEP services to a median of 400 clients (*interquartile range*: 106, 900) in the past year. Among the 37 clinical organizations offering same-day PrEP (*N* = 26), a median of 50 individuals (*interquartile range*: 7, 90) initiated PrEP through a same-day protocol. The 26 clinical organizations currently implementing same-day PrEP reported having done so for a median of 5 years thus far *(interquartile range*: 3, 6), and a published protocol for same-day PrEP provision was present in 65.0% of these organizations.

### Pathways to Same-Day PrEP Non-Implementation

As stated previously, 32% of the clinical organizations surveyed were not yet implementing same-day PrEP. We describe 3 CNA models, detailed in Table 3, for difference-making barriers associated with delayed implementation of same-day PrEP.

**Fig. 1:**
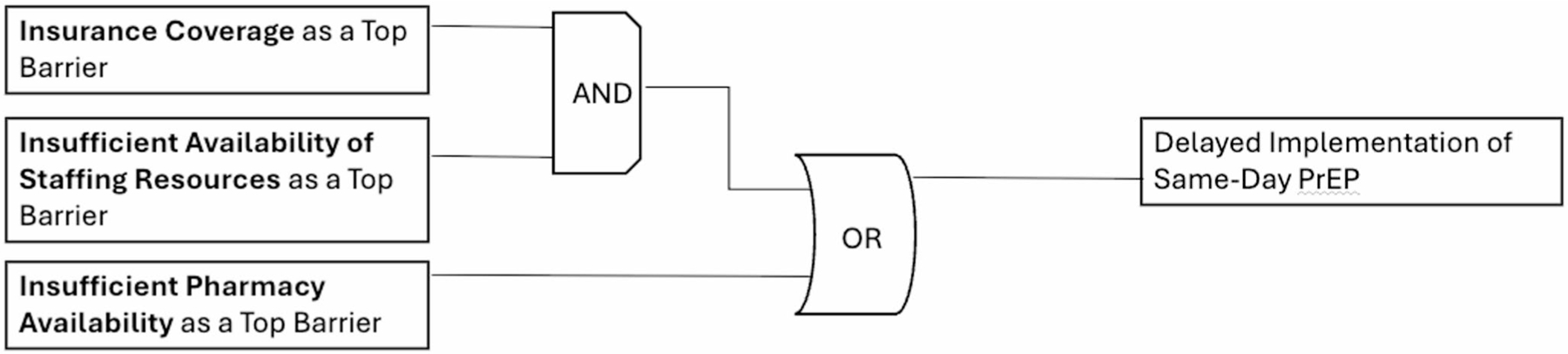
Model 1. This figure was made based on visualizations produced by Edward Miech and Deborah Cragun [46]

*Candidate Model 1* explained 50% of clinical organizations (*n* = 6/12) not currently implementing same-day PrEP with a prevalence-adjusted consistency of 85.7%. Figure 1 details this model, highlighting two discrete pathways to delayed implementation of same-day PrEP : (1) insurance coverage as a top barrier AND insufficient availability of staffing resources as a top barrier; OR (2) insufficient pharmacy availability as a top barrier.

**Fig. 2:**
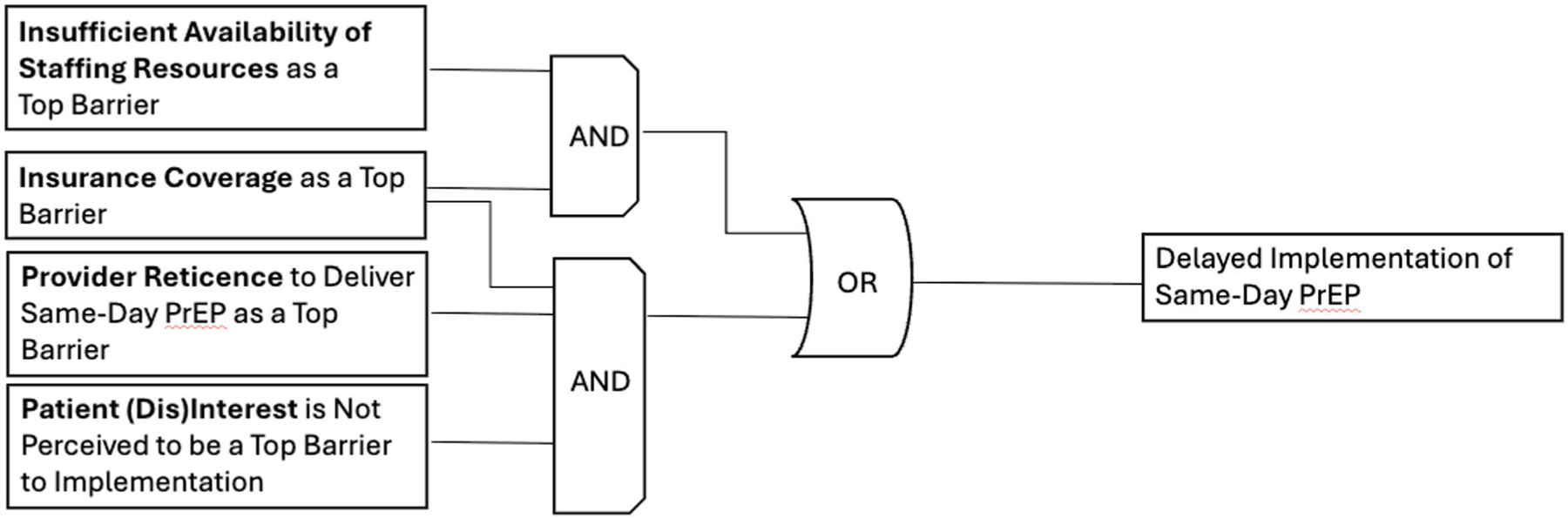
Model 2. This figure was made based on visualizations produced by Edward Miech and Deborah Cragun [46]

*Candidate Model 2* explained 50% of clinical organizations (*n* = 6/12) not currently implementing same-day PrEP, with a prevalence-adjusted consistency of 75%. Figure 2 details this model, highlighting two discrete pathways to delayed implementation of same-day PrEP: (1) insurance coverage as a top barrier AND insufficient availability of staffing resources as a top barrier; OR (2) insurance coverage as a top barrier AND provider reticence to implement same-day PrEP as a top barrier AND *patient (dis)interest in same-day PrEP not being ranked as a top barrier.*

**Fig. 3:**
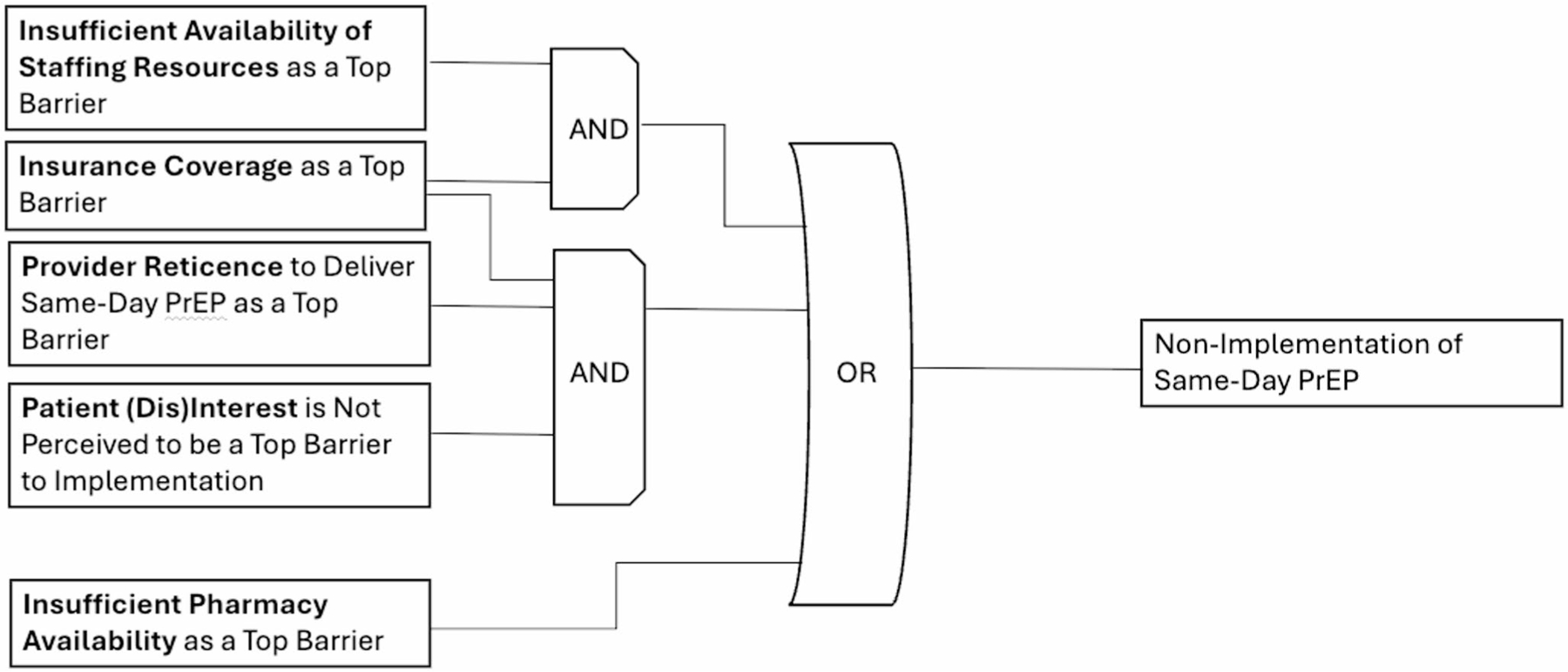
Model 3. This figure was made based on visualizations produced by Edward Miech and Deborah Cragun [46]

*Candidate Model 3* represents a combination of candidate models 1 and 2, explaining nearly 67% of clinical organizations (*n* = 8/12) not currently implementing same-day PrEP with a prevalence-adjusted consistency of 80%. Figure 3 highlights three discrete pathways to delayed implementation of same-day PrEP: (1) insurance coverage *as a top barrier* AND insufficient availability of staffing resources as a top barrier; (2) insufficient pharmacy availability as a top barrier; OR (3) provider reticence to implement same-day PrEP as a top barrier AND insurance coverage *as a top barrier* AND tpatient (dis)interest in same-day PrEP not being ranked as a top barrier. We selected Model 3 as the final model due to its higher prevalence-adjusted contrapositive coverage (0.667), high prevalence-adjusted consistency (0.80), inclusive configurations representing heterogeneous pathways to delayed implementation of same-day PrEP, and additional validation from expert member checking with the study principal investigators (PIs). Our selection of Model 3 was confirmed by the study PIs, who have intimate knowledge of real-world PrEP implementation.

As Model 3 is a combination (or supermodel) of Candidate Models 1 and 2, we examined contextual differences between the 37 clinical organizations covered by only one (but not both) Candidate Models 1 and 2. Four of the 12 clinical organizations (cases 2, 20, 25, and 41) not currently implementing same-day PrEP were covered by only Candidate Model 1 or Candidate Model 2 (but not both). We analyzed whether there were differences by clinic type (e.g., FQHC versus CBO), number of employees licensed to prescribe ART and PrEP, number of individuals provided HIV prevention and treatment services in the past year, types of services available (i.e., PrEP, ART), and the racial and gender composition of patient populations. We did not identify any discernable differences in characteristics. The sole difference across these four clinical organizations was state location; the two clinical organizations covered by Candidate Model 1 but not Candidate Model 2 were in California, while the two clinical organizations covered by Candidate Model 2 but not Candidate Model 1 were in Alabama.

As part of our analysis, we examined the three clinical organizations (cases 9, 34, and 45) that did not fit any of the 3 candidate models. We attempted to identify whether any patterns existed to explain why these three clinical organizations were not covered by our candidate models. However, we were unable to identify any distinguishing factors or patterns to explain this or hypothesize as to why it may be the case. The only factor these three clinical organizations all shared was that they served more patients for HIV treatment than for HIV prevention; however, this was not uncommon among the other cases in our analysis.

## Discussion

This manuscript contributes to a growing body of literature examining the implementation of same-day PrEP in the U.S. Notably, from our assessment of the literature, our study is the first to use CNA to identify key barriers to same-day PrEP implementation. Identifying these barriers is an important first step to selecting, developing, and trialing implementation strategies. The three identified pathways to delayed implementation of same-day PrEP demonstrate that barriers in some clinics may not be equally hindering in others. Thus, strategies must be tailored to implementation contexts, specifically the policy context, as discussed below.

One identified pathway to delayed implementation of same-day PrEP included insurance coverage challenges and insufficient staffing resources as top barriers. These two barriers, considered together, highlight policy and financing complexities related to same-day PrEP implementation, impacting clients and the health workforce. Indeed, recent studies examining the effectiveness of same-day PrEP implementation have found that many patients require financial assistance to offset PrEP-related out-of-pocket expenses [19–22]. Another study of same-day PrEP implementation in a clinic with an onsite pharmacy attributed the sustainability of implementation to the pharmacy’s capacity to bill insurance plans; this capacity hinged upon an enabling local legislative environment [47]. Federal policy changes in 2024 advanced patients’ ability to access no-cost PrEP, including the elimination of co-payments through Medicaid expansion. States like California and Illinois also have state PrEP assistance programs that cover the cost of PrEP, regardless of insurance status [48, 49]. Thus, policy-level strategies are needed to expand access to PrEP.

Another identified pathway to delayed implementation of same-day PrEP included the following: insurance coverage barriersand provider reticence to implement same-day PrEP as top barriers, as well as patient (dis)interest in same-day PrEP not being ranked as a top barrier. Interestingly, two Alabama clinical organizations were covered by this pathway but not by the third (i.e., insurance coverage barriers and insufficient staffing resources). Alabama is one of ten states in which Medicaid was not expanded after the implementation of the Affordable Care Act, which broadened eligibility to include all families making less than 138.0% of the federal poverty threshold [50]. This change in eligibility expanded access to care for low-income individuals, increasing coverage for those who are not parents by an average of 10.5% [51]. As a result, studies have found that Medicaid expansion is associated with increased PrEP use, particularly in urban areas [52–55]. It is possible that insurance navigation complexities and provider discomfort in prescribing PrEP in the absence of confirmatory HIV testing also disincentivize same-day PrEP implementation at the clinic level. Thus, perceived patient demand (or lack thereof) for same-day PrEP would insufficiently influence same-day PrEP implementation within these clinics. Our prior research with health department leaders found a perception that the funding environment in Alabama made it difficult for providers to prioritize expanding new services or improving existing ones [24, 31]. It would make sense then, that providers may not know whether there is patient interest in same-day PrEP. If providers do not discuss same-day PrEP with patients or feel uncomfortable prescribing PrEP without conducting confirmatory testing, patients may not express preferences for same-day PrEP [22]. In comparison, patients in California have access to a state drug assistance program for PrEP, which covers PrEP-related costs even for patients with insurance coverage [48].

The third and final pathway to delayed implementation of same-day PrEP was insufficient pharmacy availability alone. Prior research has identified clinics’ ability to provide rapid access to medication as an important facilitator of same-day PrEP implementation [17, 20, 22]. Having an onsite pharmacy enables clinics to provide PrEP starter packs for oral PrEP and to facilitate same-day initiation of LAI PrEP for patients whose insurance plans require PrEP prescriptions to be filled by a pharmacy [17, 22]. Research examining pharmacist-led same-day PrEP has identified preliminary successes with same-day prescriptions but less success with linking patients to ongoing care [23]. By integrating pharmacy services into clinics, onsite pharmacies could bridge this gap between uptake and adherence. Currently, pharmacists in at least 17 states can initiate and prescribe PrEP due to legislation, such as Illinois’ House Bill 4430, and it is possible for pharmacists in other states to enter into collaborative practice agreements (CPAs) with medical providers, enabling pharmacists to order laboratory examinations, provide PrEP counseling, dispense medication, and refill prescriptions [56, 57]. Alternative strategies may also include delivering medications to the patient’s residence. Recent domestic and international research has identified mail-delivered PrEP as highly acceptable, more preferred than receiving PrEP at a clinic or pharmacy, and a potential vehicle for mitigating PrEP stigma [58–60]. Various models of remotely delivered or “TelePrEP” programs have been investigated, including differing combinations of (1) in-person laboratory testing or mailed-in HIV/STI testing but in-person laboratory processes for other diagnostic purposes (e.g., creatinine testing); (2) virtual PrEP navigators, in-person case managers/PrEP navigators or text messaging/phone calls for support; and (3) mail delivery of PrEP [61]. However, these alternative service delivery platforms will not overcome PrEP-related implementation constraints across settings, as those who are unhoused will likely need to continue picking up prescriptions or receiving LAI PrEP injections in person.

Although our analysis is focused on the key barriers that explain delayed implementation of same-day PrEP, our team has elsewhere descriptively examined the clinical organizations currently implementing same-day PrEP [31]. A majority of these (76.7%) were in Medicaid expansion states. Our team identified top, self-reported facilitators to same-day PrEP, as well, including increased funding, provider training, and mechanisms to provide care regardless of insurance status. Those findings confirm our own suggestions here in this discussion to increase funding, implement tele-PrEP options, and bolster Medicaid coverage.

While our analysis is focused on provider-identified barriers, researchers and implementers must consider patients’ perspectives. Prior research has found same-day PrEP to be highly acceptable among patients being offered same-day services, with patients noting that the ease of having a single visit to initiate PrEP reduces barriers to initiation [17]. As noted above, PrEP navigators can also facilitate PrEP initiation and greater individual comfort and self-efficacy with PrEP self-management. Several studies of PrEP users have found that their perceived susceptibility to HIV evolved after PrEP initiation, resulting in eventual discontinuation [20, 23]. Navigators can help assist patients in navigating the dynamic “seasonality” or “cycles” of HIV risk that may warrant PrEP [62, 63]. Further study of PrEP users’ experiences with same-day initiation is critical to identifying patient-level barriers to same-day PrEP implementation. Additionally, there is a need to implement and expand efforts by the CDC to award funding to health departments to cover the costs of patient navigators and case managers (PS24-0047). Most recently, the Supreme Court of the United States ruled in favor of provisions of the Affordable Healthcare Act that mandate insurance plans to cover PrEP at no cost—a ruling that will maintain affordable access to PrEP care for this with insurance [64].

To further reduce disparities in access to care, there is a need for increased attention to public and private-sector financing of PrEP. Medicaid provides financial stability to hundreds of community health centers and hospitals across the nation, reduces the amount of care healthcare organizations provide without compensation, and provides healthcare coverage to millions of patients [65, 66]. When patients lose access to insurance coverage, this not only limits their ability to access care, but it also leaves healthcare organizations in a dire predicament. Researchers have found that “each newly uninsured person leads to nearly $900 in uncompensated care costs” [67]. As a result of hospitals and healthcare organizations absorbing these costs, fewer providers, programs, and services become available for all patients [66]. Further, the flexibility states are granted to cover costs to address and mitigate social determinants of health can increase access, uptake, and adherence to evidence-based interventions, like PrEP, for marginalized and low-income populations [68]. In states like Illinois, for example, Medicaid provides funding for housing support, violence prevention interventions, community reintegration from criminal legal facilities, food and nutrition services, and employment assistance [69]. Addressing social determinants of health through increased funding for social interventions (e.g., housing assistance, patient navigation) ultimately reduces costs of care for healthcare organizations and patients [70]. Strategies for researchers and providers to encourage policymakers to bolster funding, demystify “black-box” insurance processes (e.g., pre-authorization processes, claims denials), and to encourage pharmaceutical companies to expand patient assistance programs may include translating research evidence for policymakers, pharmaceutical companies, and the larger public, forming broad coalitions, and even grassroots activism, particularly with the passage of H.R.1, also known as (“One Big Beautiful Bill,”) which is estimated to strip insurance coverage from nearly 17 million people over the next decade [71–73]. This study should be considered with the following limitations in mind. First, we examined factors associated with delayed implementation of same-day PrEP. While we can posit that strategies targeting these factors may facilitate implementation, we did not explicitly examine which enabling factors or strategies lead to implementation. Second, the data had a high degree of imbalance regarding the outcome. This is likely due to the construction of the survey instrument, as only clinical organizations currently implementing same-day PrEP or those planning to do so in the future were asked questions about barriers and facilitators to implementation. CNA is most robust when the data—particularly the outcome–have greater balance (generally a 60/40 split at minimum). When the distribution in the outcome is skewed, it is difficult for the algorithm to identify factors that explain an outcome. As such, we used prevalence-adjusted consistency and prevalence-adjusted contrapositive coverage thresholds, which enabled us to identify paths to delayed implementation. With a more balanced outcome, these paths may differ. Additionally, our survey questions relied on self-report at the clinic level; in addition to leading to possible information bias, we did not seek clarity on the routinization of same-day PrEP practices into care. Thus, it is possible that within the clinics there is high heterogeneity across providers in the implementation of same-day PrEP that these data do not capture Finally, the lower consistency thresholds of all three models signify that there may be some “noise” in our candidate models. Nonetheless, our study highlights factors that differentiate clinical organizations not currently implementing same-day PrEP from those currently implementing same-day PrEP (e.g., pharmacy access, staffing resources, insurance coverage). These findings highlight future directions for researchers to explore in greater detail.

## Conclusion

Same-day PrEP is a novel evidence-based practice that may reduce some barriers to HIV prevention access, including challenges associated with attending multiple appointments, suboptimal transportation access, and communication loss during the linkage-to-care process. Our study builds on prior literature that has qualitatively identified barriers to same-day PrEP implementation (e.g., patient-centeredness, PrEP/peer navigation, shifting workflows) [17, 19–23].

While this prior body of research has identified barriers and facilitators within clinics, these determinants rarely act in isolation [74]. Further, it is not always the case that there is a one-size-fits-all pathway to implementation success. CNA aids this process by identifying multiple configurations of factors that explain delayed implementation of same-day PrEP. In the present study, we identified key barriers to same-day PrEP implementation, primarily clustering in the domains of resource restrictions (e.g., limited staffing resources, absence of onsite pharmacies) and financial/policy complexities (e.g., insurance coverage complexities). The next step in this process is to reverse map and/or co-develop implementation strategies based on our candidate models and informed by ongoing interviews with patients and prior interviews with high-level jurisdictional leaders [75]. We plan to share these results back with the clinical organizations that participated in our survey. These organizations can similarly reverse-map implementation strategies using free and user-friendly tools like the StrategEase tool [76].

## Supplementary Material

Supplementary Material 1

**Supplementary Information** The online version contains supplementary material available at https://doi.org/10.1007/s10461-025-04898-2.

## Figures and Tables

**Table 1:** Data dictionary

Variable^1^	Definition^2,3^
LEADERSHIP	Insufficient leadership buy-in and support to implement same-day PrEP
PROVIDER COMFORT	Provider discomfort with implementing same-day PrEP
PROVIDER INTEREST	Lack of provider interest in implementing same day PrEP.
PROVIDER CAPABILITY	Providers do not feel they have the awareness, knowledge, and/or training to implement same day PrEP.
INSURANCE	Insurance coverage is a barrier to implementing same day PrEP.
RESOURCES	Insufficient availability of staffing resources for implementing same-day PrEP
WORKFLOW	Existing clinic workflows are not sufficient to implement same day PrEP.
PRIORITIES	Same-day PrEP is of a lower priority to other programs and services currently being implemented
PATIENT INTEREST	Insufficient patient interest to implement same-day PrEP
PSYCH NEEDS OF PATIENTS	There is insufficient support for the psychological needs of patients to implement same-day PrEP
PHARMACY	There are no on-site medications available and/or no collaborating pharmacy to implement same day PrEP.
PHLEBOTOMY/LABS	There is no on-site phlebotomy and/or laboratory to implement same day PrEP.
PROTOCOL	There are no existing protocols or guidelines within the clinic or system for implementing same-day PrEP

1 The outcome, *DELAYED IMPLEMENTATION OF SAME-DAY PREP*, was defined as the clinic not implementing same-day PrEP at present but having intentions to do so within the next 12 months

2 All variables were coded as “0” referring to the barrier not being endorsed by the clinic (i.e., the barrier was not perceived to be a top barrier to implementing same-day PrEP) and “1” referring to the barrier being endorsed by the clinic (i.e., the barrier was perceived to be a top barrier to implementing same-day PrEP)

3 Respondents were asked to note whether the determinant was a barrier to implementing same day PrEP on a Likert scale of 0 to 3, with 0 referring to the absence of the barrier and 3 referring to the barrier as present and highly impacting same day PrEP implementation. To prevent extreme data imbalances, we coded any endorsement of the determinant as a barrier to implementation as “1”

**Table 2:** Demographics and clinic characteristics

Characteristic (*n*, %)	Clinics (*N* = 38)^1^
Clinic type^#^	
Federally qualified health center (FQHC)	14 (37.0%)
Academic-affiliated health center	10 (26.0%)
Hospital-based clinic	8 (21.0%)
Community-based organization clinic	7 (18.0%)
Public health clinic	4 (11.0%)
Private (for-profit) clinic	1 (2.6%)
Clinic jurisdiction	
Los Angeles	8 (21.0%)
San Diego	6 (15.8%)
Chicago	8 (21.0%)
Alabama	6 (15.8%)
Dallas/Tarrant	4 (10.5%)
Baltimore	6 (15.8%)
Number of employees providing HIV-related services (median, IQR), *n* = 36	23 (17, 50)
Missing	2
Number of employees licensed to prescribe ART and PrEP (median, IQR), *n* = 36	7 (5, 14)
Missing	2
Geographic coverage of HIV service delivery	
Urban only	25 (66.0%)
Rural only	2 (5.3%)
Both rural and urban	12 (32.0%)
Number of PrEP initiations in the past year (median, IQR), *n* = 36	400 (106, 900)
Number of years implementing same-day PrEP services (median, IQR), *n* = 24*	5 (3, 6)
Number of same-day PrEP initiations in the past year (median, IQR), *n* = 24*	50 (7, 90)
Same-day PrEP implementation protocol available, *n* = 26*	17 (65%)

1 Median (Q1-Q3); n(%);

* Among clinics reporting current implementation of same-day PrEP services (*N* = 26)

# Clinics may respond for more than one clinic type

**Table 3:** CNA Models^1^

Clinic	Delayed implementation of same day PrEP	Provider reticence	Insurance-Coverage barriers	Insufficient availability of resources	Patient (Dis)Interest	Pharmacy inavailability	Lack of protocol	M1: INSURANCEBARRIERS* INSUFFICIENT AVAILABILITYRESOURCES	M2: PROVIDER RETICENCE* INSURANCE *patient disinterest	M3: PHARMACYINAVAILABILITY	S1: M1 + M3^2^	S2: M1 + M2^3^	S3: M1 + M2 + M3^4^
2	1	1	0	1	0	1	0	0	0	1	1	0	1
9	1	0	1	0	1	0	0	0	0	0	0	0	0
16	1	0	1	1	1	0	0	1	0	0	1	1	1
20	1	1	1	0	0	0	0	0	1	0	0	1	1
24	1	0	1	1	0	0	1	1	0	0	1	1	1
25	1	1	1	0	0	0	0	0	1	0	0	1	1
30	1	0	1	1	0	0	0	1	0	0	1	1	1
34	1	0	0	1	0	0	0	0	0	0	0	0	0
35	1	0	1	1	0	1	0	1	0	1	1	1	1
37	1	0	0	1	1	0	0	0	0	0	0	0	0
41	1	0	0	0	0	1	1	0	0	1	1	0	1
45	1	0	0	0	0	0	0	0	0	0	0	0	0
3	0	1	0	1	0	0	0	0	0	0	0	0	0
4	0	0	0	1	0	0	0	0	0	0	0	0	0
5	0	0	0	0	0	0	1	0	0	0	0	0	0
7	0	1	1	0	1	0	0	0	0	0	0	0	0
8	0	0	0	0	1	0	0	0	0	0	0	0	0
12	0	0	0	1	0	0	0	0	0	0	0	0	0
14	0	0	0	1	1	1	0	0	0	1	1	0	1
15	0	0	0	0	0	0	0	0	0	0	0	0	0
17	0	0	0	0	0	0	0	0	0	0	0	0	0
18	0	0	0	0	0	0	0	0	0	0	0	0	0
19	0	1	0	0	0	0	1	0	0	0	0	0	0
22	0	0	1	1	0	0	0	1	0	0	1	1	1
26	0	1	0	0	0	0	0	0	0	0	0	0	0
29	0	1	0	0	0	0	0	0	0	0	0	0	0
38	0	1	1	0	1	0	0	0	0	0	0	0	0
40	0	1	0	0	0	0	0	0	0	0	0	0	0
43	0	1	0	0	0	0	0	0	0	0	0	0	0
44	0	0	0	0	1	0	0	0	0	0	0	0	0
48	0	0	0	0	1	0	0	0	0	0	0	0	0
49	0	0	1	0	1	0	0	0	0	0	0	0	0
50	0	0	1	0	0	0	0	0	0	0	0	0	0
51	0	1	0	0	1	0	0	0	0	0	0	0	0
53	0	0	1	0	0	0	0	0	0	0	0	0	0
54	0	0	1	0	1	0	0	0	0	0	0	0	0
55	0	0	0	1	0	0	1	0	0	0	0	0	0
56	0	0	1	0	1	0	0	0	0	0	0	0	0

1 In CNA models, the Boolean operator “+” refers to OR and “*” refers to AND. M refers to a minimally sufficient condition, and S refers to a candidate model. Factors in all capital letters signify the presence of those factors in a solution. Factors in all lowercase letters signify the absence of those barriers in a solution

2 Normalized fit-robustness score: 0.595; Prevalence-Adjusted Consistency: 0.857; Prevalence-Adjusted Contrapositive Coverage: 0.5

3 Normalized fit-robustness score: 0.486; Prevalence-Adjusted Consistency: 0.75; Prevalence-Adjusted Contrapositive Coverage: 0.5

4 Normalized fit-robustness score: 0.892; Prevalence-Adjusted Consistency: 0.8; Prevalence-Adjusted Contrapositive Coverage: 0.667
