# Supplementary material for: Barriers to Same-Day Pre-exposure Prophylaxis (PrEP) Implementation in Federally Funded HIV Clinics Within High-Burden Areas of the U.S.: A Coincidence Analysis
Source: AIDS Behav. 2025 Oct 10;30(2):558–70. doi: 10.1007/s10461-025-04898-2 (PMC12765568; doi:10.1007/s10461-025-04898-2)
Supplement: Supplementary file 1 — Supplementary Material 1 [file 10461_2025_4898_MOESM1_ESM.docx]

**Appendix 1 – R Script**

# clean environment

rm(list =ls())

cat("\014")

# packages & sources

library(tibble); library(readr); library(QCA); library(SetMethods)

library(cna); library(readxl); library(tidyverse); library(dplyr)

library(lifecycle); library(frscore); library(openxlsx); library(cnaOpt)

library(causalHyperGraph)

# set working directory

setwd("/Volumes/Memorex USB/Projects/CNA/CNA_Analyses")

dt1<-Multisite_001_Data_Input

#### msc ####

names(dt1)

dim(dt1)

table(dt1)

msc<-msc(mvcna(dt1,ordering=list("RAPIDPREP_NO"),

strict=TRUE, suff.only=TRUE, maxstep=c(3,4,10)))

### rearrange

msc<-arrange(msc, desc(outcome), complexity, desc(coverage), desc(consistency))

#---- export list

wb <- createWorkbook()

addWorksheet(wb, "msc_multisite")

writeData(wb, "msc_multisite", msc)

saveWorkbook(wb, "msc_multisite.xlsx", overwrite = TRUE)

## stopped here to see which rise to the top

#### CNA ####

names(dt1)

table(dt1)

summary(dt1)

cna(dt1, outcome ="RAPIDPREP_NO")

cna(dt1, con = .95, cov = .95, outcome = "RAPIDPREP_NO")

cna(dt1, con = .95, cov = .9, outcome = "RAPIDPREP_NO")

cna(dt1, con = .9, cov = .9, outcome = "RAPIDPREP_NO")

cna(dt1, con = .9, cov = .85, outcome = "RAPIDPREP_NO")

cna(dt1, con = .85, cov = .85, outcome = "RAPIDPREP_NO")

cna(dt1, con = .85, cov = .80, outcome = "RAPIDPREP_NO")

cna(dt1, con = .80, cov = .80, outcome = "RAPIDPREP_NO")

cna(dt1, con = .80, cov = .75, outcome = "RAPIDPREP_NO")

cna(dt1, con = .75, cov = .75, outcome = "RAPIDPREP_NO")

#### PREVALENCE ADJUSTED CONSISTENCY AND COVERAGE ####

remove.packages("cna")

install.packages("cna_3.5.3.4.tar.gz", repos = NULL, type ="source")

library(cna)

stopifnot(packageVersion("cna") == "3.5.3.4")

def_w_measures <- ccDef_ratio(list(

cbind(c(1, 0, 0, 0, 0, 0),

c(1, 0, -99-1i, 0, -99-1i, 0)),

cbind(c(0, 1, 0, 0, 0, 0),

c(0, 1, 0, -99, -99, 0))))

cna(dt1, con = .95, cov = .95, outcome = "RAPIDPREP_NO", ccDef = def_w_measures)

cna(dt1, con = .80, cov = .80, outcome = "RAPIDPREP_NO", ccDef = def_w_measures)

cna(dt1, con = .80, cov = .75, outcome = "RAPIDPREP_NO", ccDef = def_w_measures)

cna(dt1, con = .75, cov = .75, outcome = "RAPIDPREP_NO", ccDef = def_w_measures)

cna(dt1, con = .75, cov = .70, outcome = "RAPIDPREP_NO", ccDef = def_w_measures)

cna(dt1, con = .70, cov = .70, outcome = "RAPIDPREP_NO", ccDef = def_w_measures)

library(frscore); library(cnaOpt)

x <- rean_cna(dt1, outcome="RAPIDPREP_NO",

strict=F,attempt = seq(.8, .5, -0.1), output="asf",

ccDef = def_w_measures)

M <- do.call(rbind, x)

##fr score

s1<-frscore(M$condition, dat=dt1)

s1

#------- combine outputs from cna, frscore

y <- condTbl(s1$models$model, dt1)

# round frscore to 3 decimals

s1$models$norm.score <- round(s1$models$norm.score, digit=3)

s1$models$con <- round(y$consistency, digit=3)

s1$models$cov <- round(y$coverage, digit=3)

s1$models$complex <- round(y$complexity, digit=3)

s1$models$fit2<-round(y$consistency*y$coverage, digit=3)

# add spaces around '+'

s1$models$model<-gsub("+", " + ", s1$models$model, fixed=TRUE)

#---- export list

wb <- createWorkbook()

addWorksheet(wb, "asf_9Xs")

writeData(wb, "asf_9Xs", as.data.frame(s1$models))

saveWorkbook(wb, "out_RAPIDPREP_NO.xlsx", overwrite = TRUE)
